# Reinfection of Nine-Valent Human Papillomavirus Vaccine Types Among HIV-Negative Men Who Have Sex With Men: A Prospective Cohort Study

**DOI:** 10.3389/fpubh.2022.896479

**Published:** 2022-07-18

**Authors:** Lirong Liu, Guozhen Zhang, Zewen Zhang, Lu Wang, Duolao Wang, Jianghong Dai

**Affiliations:** ^1^Department of Epidemiology and Biostatistics, School of Public Health, Xinjiang Medical University, Urumqi, China; ^2^Center for Disease Control and Prevention of Yining, Yining, China; ^3^Department of Clinical Sciences, Liverpool School of Tropical Medicine, Liverpool, United Kingdom

**Keywords:** reinfection, human papillomavirus, men who have sex with men, nine-valent vaccine-preventable HPV, HIV-negative

## Abstract

**Objectives:**

Reinfection of human papillomavirus (HPV) among men who have sex with men (MSM) after clearing the infection of HPV has not been well characterized. To understand the reinfection of HPV among human immunodeficiency virus (HIV) negative MSM without HPV vaccine, we analyzed the reinfection of nine-valent HPV vaccine (9v-HPV) types.

**Methods:**

Data were acquired from a prospective cohort study among HIV-negative MSM in Urumqi of Xinjiang from 1 April 2016 to 14 July 2020. Participants were recruited through a local non-government organization using a website advertisement. Self-administered questionnaires and self-collected anal swabs were collected at baseline and every 6 months. The incidence rates of reinfection was calculated based on the number of events divided by person-months of observation of event risk. 95% confidence intervals (*CIs*) were calculated based on the Poisson distribution.

**Results:**

A total of 515 HIV-negative unvaccinated MSM were included with a mean age of 30.93 years (*SD* 7.85), and 27.68% were reinfected with any 9v-HPV type after clearance. The reinfection incidence rate of any 9v-HPV was 14.47 per 1000 person-months (95% *CI*: 11.52–17.87). HPV52 was the most common type of reinfection, with a reinfection rate of 17.96 per 1,000 person months (95% *CI*: 11.58–26.33). Univariate analysis showed that MSM over the age of 30 had a slightly higher risk of reinfection with any 9v-HPV (Hazard ratio (*HR*): 1.57; 95% *CI*: 1.01–2.45), but no significant association was observed in multivariate analysis.

**Conclusions:**

Our study showed MSM without HPV vaccine will become reinfected following the natural clearance of specific HPV types. It is also suggested that HPV vaccination is recommended not only prior to sexual debut but also after viral clearance for MSM to reduce HPV prevalence.

## Introduction

Human papillomaviruses (HPVs) are one of the most common sexually transmitted viruses in men and women. Because of their frequent high-risk sexual behaviors, men who have sex with men (MSM) are at increased risk for anal HPV infection and HPV-related diseases (1–3), while persistent infection might induce precancerous lesions and cancers (4). The estimated prevalence of anal HPV among human immunodeficiency virus (HIV) negative MSM in China was 53.6%, and the most frequently detected HPV genotypes were HPV6, HPV16, HPV11, HPV52, and HPV58 (5–7). Several prophylactic HPV vaccines have been available since 2006, such as HPV-16/18 bivalent vaccine (Cervarix^®^, GSK, and Cecolin^®^, Xiamen Innovax), HPV-6/11/16/18 quadrivalent vaccine (Gardasil^®^, Merck), and HPV-6/11/16/18/31/33/45/52/58 nine-valent vaccine (Gardasil ^®^9, Merck). However, in many countries, including China, vaccination of HPV is still primarily directed at women (8).

Host immune responses are likely to be a critical mechanism for preventing, controlling, and eliminating HPV infection (9). However, because antibody concentrations after prophylactic HPV vaccination are higher than those observed after natural HPV infection (10), whether these naturally acquired antibodies protect against future infections has been debated in earlier studies (11–13). Studies on the natural history of HPV infection among women have shown that individuals may be susceptible to redetection of HPV infection with the same HPV type (14–16). The reinfection of HPV is an important component of the natural history and may help to elucidate the reasons for sustained genital HPV prevalence. But to our knowledge, reinfection of anal HPV among MSM is still not well characterized. More insight into the reinfection of HPV among MSM can broaden the history of HPV natural infection. Moreover, studies have shown that up to half of the HPV infections clear within 6 months, and the great majority clear within a few years after the acquisition. Because MSM cannot receive benefits from the HPV vaccine at present, understanding the reinfection of HPV among HIV-negative unvaccinated MSM can help design national vaccination programs or targeted prevention strategies.

In this prospective cohort study, we analyzed the reinfection of anal 9 HPV vaccine types (HPV-6/11/16/18/31/33/45/52/58) among HIV-negative MSM without HPV vaccine in Urumqi, China. Our objective was to estimate reinfection of anal nine-valent HPV vaccine (9v-HPV) types of MSM.

## Methods

### Study Design and Participants

We have established a dynamic prospective cohort study of HPV research among HIV-negative MSM in Urumqi, Xinjiang, China from 1 April 2016. Participants were recruited through a local non-government organization (Xinjiang Dream Health Service Center) using website advertisement, a WeChat group (an extremely popular social application), and peer recommendations. Men were eligible to participate if they (1) were at least 18-years-old; (2) self-reported having sex with men in the past 6 months; and (3) were HIV-negative.

Study visits took place every 6 months. Participants completed a self-administered questionnaire at baseline and each follow-up visit, designed to collect information on sociodemographic and sexual behavior characteristics. Participants also self-collected anal swabs and then placed them into a 3 ml sample transport medium for testing. To improve the accuracy of sampling, volunteers from non-government organization were trained by clinicians before the study, and volunteers then instructed the participants to collect anal specimens during the study. All participants were compensated Chinese ¥40 (~$6.6) at each visit.

MSM, who had received no previous dose of HPV vaccine and attended at least three visits by 14 July 2020 were included in this study. The participants provided written informed consent. This study was approved by the ethics committee of the First Affiliated Hospital of Xinjiang Medical University (20160512-11).

### HPV DNA Testing

Hybribio 37 HPV GenoArray Diagnostic Kit Test (Hybribio Biotech, Chaozhou, China) was used for HPV detection and genotyping. Details of HPV DNA testing methods were published elsewhere (17). Briefly, a polymerase chain reaction (PCR) HPV genotyping test (Hybribio Biotech Limited Corporation, Chaozhou, China) was used to determine the HPV-type distribution in the anal swab samples. The PCR test amplified the target HPV DNA for 37 types of HPV, which included HPV6, 11, 16, 18, 26, 31, 33, 34, 35, 39, 40, 42, 43, 44, 45, 51, 52, 53, 54, 55, 56, 57, 58, 59 61, 66, 67, 68, 69, 70, 71, 72, 73, 81, 82, 83, and 84. A quality-controlled template DNA probe was used for amplification and as an internal control in each experiment.

### Variables and Definitions

Sociodemographic variables of interest included age, education, registered residence, ethnicity, marital, employment status, and average monthly income. Sexual behaviors included age of first anal intercourse, self-reported sexual orientation, the gender of sexual partners, experience of anal intercourse in the past 6 months, pattern of anal intercourse in the past year, number of sexual partners in the past 6 months, number of anal sex encounters in the last week, drug use, STD infection in the past year, smoking status, and alcohol consumption.

To study reinfections with a given HPV type, clearance of the infection was defined as a negative test result of the same HPV type after the positive detection of an HPV type (1-0). The reinfection was defined as the positive detection again of the same HPV type after being cleared of an HPV type (1-0-1). In a sensitivity analysis, we used a conservative definition of clearance that required at least two consecutive negative samples (1-0-0). This stricter definition is evaluated by considering that a single negative visit between two positives may be due to the DNA content in the sample being below the threshold of detectability. Clearance of bivalent HPV (2v-HPV) types of infection was defined as a negative test result of both HPV16 and HPV18 after the positive detection of HPV16 or/and HPV18. Reinfection of any 2v-HPV was defined as the positive detection again of HPV16 or/and HPV18 after clearance of 2v-HPV types. Any quadrivalent HPV (4v-HPV) type (HPV6/11/16/18) and any 9v-HPV (HPV6/11/16/18/31/33/45/52/58) data were defined in the same way.

### Statistical Analyses

Descriptive statistics included the mean and standard deviation (*SD*) or median and interquartile range (*IQR*) for continuous variables, frequency, and percentages for categorical variables. The incidence rates of reinfection, including 95% confidence intervals (*CIs*), were calculated based on the number of events divided by the time of event risk observed. The time to risk of reinfection events was the time in a month from the first clearance to the date that the subject first tested re-positive for a given HPV type in subsequent follow-up. Each HPV infection was treated as an independent event in the study. The 95% *CIs* were calculated based on the Poisson distribution. A Cox proportional hazards model was used to analyze the factors associated with reinfection. The variables with *p* < 0.20 in the univariate analyses and the influencing factors reported in the literature were included in the multivariate Cox proportional hazards model to determine the factors associated with HPV reinfections. Data analysis was performed using R version 4.0.2.

## Results

**Figure 1:**
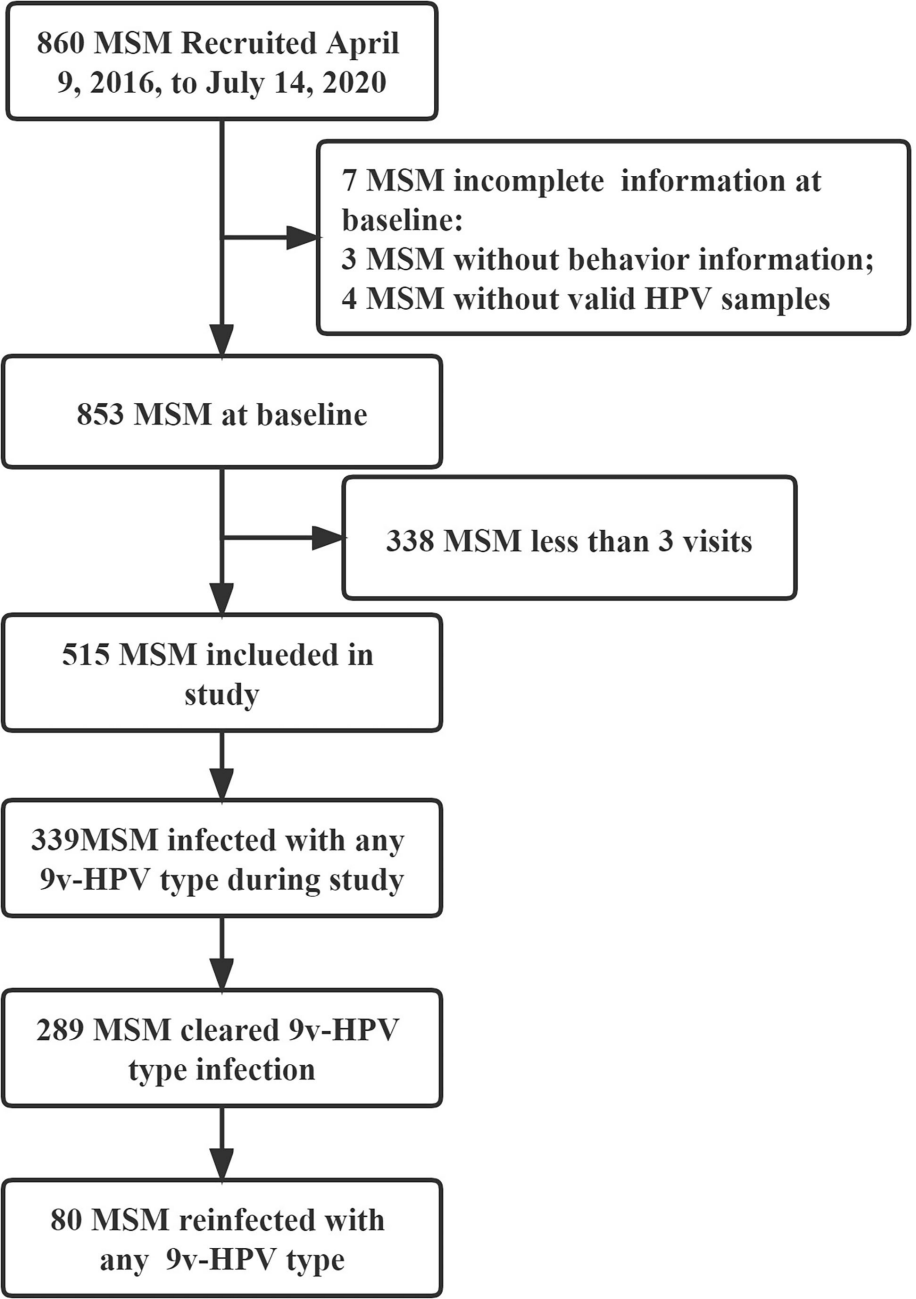
Flowchart of human immunodeficiency virus (HIV)-negative men who have sex with men (MSM) for the study. MSM, men who have sex with men; HIV, human immunodeficiency virus; HPV, human papillomavirus; 9v-HPV, nine-valent HPV types, such as HPV6, 11, 16, 18, 31, 33, 45, 52, and 58.

Between 9 April 2016, and 14 July 2020, a total of 860 HIV-negative MSM without HPV vaccine were recruited in the study, of whom 515 provided at least three visits with qualified samples for HPV testing included in the study (4 without valid samples for HPV testing at baseline, 3 without a behavior information questionnaire at baseline, and 338 with <three visits were excluded). The median duration of follow-up was 31.50 months (*IQR* 22.77–37.80), and the median number of follow-up visits was 6 times (IQR 4–7). Among 515 MSM, a total of 393 (76.31%) had any 9v-HPV infection during the study, natural clearance occurred in 73.54% (289/393). In total, 27.68% (80/289) were reinfected with any 9v-HPV after clearance (Figure 1).

### Participant Baseline Characteristics

The mean age of the 515 MSM at enrolment was 30.93 years (SD 7.85). At baseline, a total of 396 (76.89%) identified themselves as homosexual and 409 (79.42%) reported having had the experience of anal intercourse in the past 6 months. A total of 259 (50.29%) had one and more than one sexual partner in the past 6 months and only 92 (17.86%) had one and more than one time of anal sex encounter in the last week. The demographic and sexual behaviors baseline characteristics of 515 MSM are reported in Table 1.

**Table 1:** Demographic and sexual behaviors among human immunodeficiency virus (HIV)-negative men who have sex with men (MSM) at baseline (*N* = 515).

**Variable**	**Participants, No/Mean (SD/%)**
**Age (years)**	
Mean (SD)	30.93 (7.85)
**Ethnicity**	
Han	457 (88.74)
Others	58 (11.26)
**Education level**	
High school or lower	107 (20.78)
Junior college	149 (28.93)
University or higher	259 (50.29)
**Marital status**	
Single	386 (74.95)
Married	87 (16.89)
Divorced/widowed	42 (8.16)
**Age at first insertive or receptive anal sex**	
≤ 18	159 (30.87)
>18	356 (69.13)
**Self-reported sexual orientation**	
Homosexual	396 (76.89)
Bisexual/heterosexual	119 (23.11)
**Gender of sexual partners**	
Only male	288 (55.92)
Both male and female	227 (44.08)
**Experience of anal intercourse in the past 6 months**	
Yes	409 (79.42)
No	106 (20.58)
**Pattern of anal intercourse in the past year**	
Mainly receptive	221 (42.91)
Mainly insertive	294 (57.09)
**Number of sexual partners in the past 6 months**	
≥1	259 (50.29)
<1	256 (49.71)
**Number of anal sex in the last week**	
≥1	92(17.86)
<1	423(82.14)
**Drug use**	
Yes	153 (29.71)
No	362 (70.29)
**Smoking history**	
Yes	265 (51.46)
No	250 (48.54)
**Drinking history**	
Yes	436 (84.66)
No	79 (15.34)

*MSM, men who have sex with men; HIV, human immunodeficiency virus; SD, Standard deviation*.

**Figure 2:**
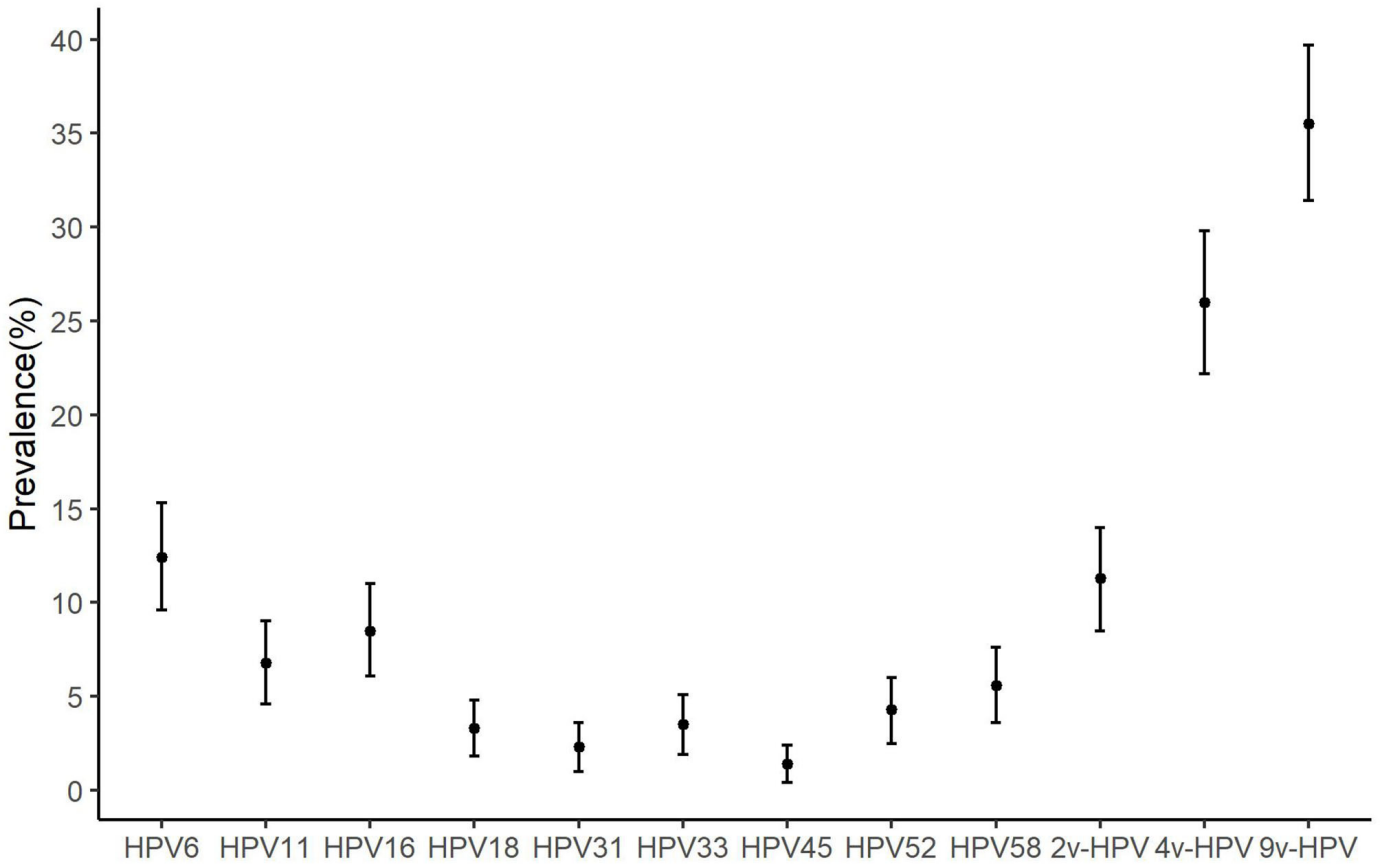
The prevalence of anal human papillomavirus (HPV) types among HIV-negative MSM. MSM, men who have sex with men; HPV, human papillomavirus; HIV, human immunodeficiency virus; Bar, 95% *CI* of prevalence; 9v-HPV, nine-valent HPV types, such as HPV6, 11, 16, 18, 31, 33, 45, 52, and 58; 4v-HPV, quadrivalent HPV types, such as HPV6, 11, 16, and 18; 2v-HPV, bivalent HPV types, such as HPV16 and HPV18.

The prevalence at baseline of any 2v-HPV, any 4v-HPV, and any 9v-HPV type was 11.26% (58/515), 26.02% (134/515), and 35.53% (183/515), respectively (Figure 2). HPV6 (64, 12.43%) had a higher prevalence, followed by HPV16 (44, 8.54%) (Figure 2).

### Reinfection Incidence Rate

The incidence rate of reinfection of any 9v-HPV was 14.47 per 1,000 person-months (95% *CI*: 11.52–17.87). The incidence rate of reinfections varied substantially by HPV type. The reinfection rate was highest for HPV52 (rate 17.96 per 1,000 person-months, 95% *CI*: 11.58–26.33) and lowest for HPV45 (rate 1.59 per 1,000 person-months, 95% *CI*: 0.99–6.99). HPV6 (26 participants) and HPV16 (25 participants) had a higher number of reinfected individuals compared to other types, the reinfection rate was 9.64 per 1,000 person-months (95% *CI*: 6.39–13.83) and 11.58 per 1,000 person-months (95% *CI*: 7.61–16.73), respectively. The reinfection incidence rate of each HPV type is shown in Table 2.

**Table 2:** Incidence rates^*^ of reinfection of human papillomavirus (HPV) among HIV-negative MSM (*N* = 515).

**Type**	**Infected number (%)**	**Cleared number (%)**	**Reinfected number (%)**	**Person-months at risk**	**Rate (95% CI)**
9v-HPV	393 (76.31)	289 (73.54)	80 (27.68)	5,529.77	14.47 (11.52–17.87)
4v-HPV	335 (65.05)	254 (75.82)	55 (21.65)	5,101.48	10.78 (8.18–13.89)
2v-HPV	185 (35.92)	136 (78.72)	11 (8.09)	3,151.95	3.49 (1.81–5.98)
HPV6	171 (33.20)	135 (78.95)	26 (19.26)	2,697.57	9.64 (6.39–13.83)
HPV11	104 (20.19)	84 (80.77)	19 (22.62)	1,686.14	11.27 (6.93–17.12)
HPV16	140 (27.18)	103 (73.57)	25 (24.27)	2,159.22	11.58 (7.61–16.73)
HPV18	73 (14.17)	54 (73.97)	6 (11.11)	1,213.17	4.95 (1.97–10.02)
HPV31	59 (11.46)	48 (81.36)	10 (20.83)	1,085.45	9.21 (4.62–16.16)
HPV33	67 (13.01)	47 (70.15)	10 (21.28)	975.01	10.26 (5.14–17.99)
HPV45	39 (7.57)	27 (69.23)	1 (3.70)	629.57	1.59 (0.09–6.99)
HPV52	102 (19.81)	73 (71.57)	23 (31.51)	1,280.80	17.96 (11.58–26.33)
HPV 58	94 (18.25)	78 (82.98)	22 (28.21)	1,470.49	14.96 (9.55–22.11)

* *Per 1,000 person-months*.

*MSM, men who have sex with men; HIV, human immunodeficiency virus; HPV, human papillomavirus; CI, confidence interval; 9v-HPV, nine-valent HPV types, including HPV6, 11, 16, 18, 31, 33, 45, 52, and 58; 4v-HPV, quadrivalent HPV types, including HPV6, 11, 16, and 18; 2v-HPV, bivalent HPV types, including HPV16 and HPV18*.

### Risk Factors of Any 9v-HPV Reinfection

Table 3 lists the factors associated with any 9v-HPV reinfection among HIV-negative unvaccinated MSM. In univariate analyses, MSM over the age of 30 had a slightly higher risk of reinfection with any 9v-HPV [Hazard ratio (*HR*): 1.57; 95% *CI*: 1.01–2.45], but no significant association was observed in the multivariate analysis.

**Table 3:** Factors associated reinfection of any nine-valent HPV types (9v-HPV) at baseline (*N* = 289).

**Variable**	**Univariate**		**Multivariate**	
	**HR (95%CI)**	***P***	**HR (95%CI)**	***P***
**Age (years)**				
≤30	Ref			
>30	1.57 (1.01–2.45)	0.045	1.35 (0.81–2.25)	0.224
**Marital status**				
Single	Ref		ref	
Married/divorced	1.30 (0.96–1.75)	0.085	1.11 (0.78–1.57)	0.573
**Self-reported sexual orientation**				
Homosexual	0.66 (0.42–1.04)	0.074	0.69 (0.43–1.10)	0.118
Bisexual/heterosexual	Ref		ref	
**Experience of anal intercourse in the past 6 months**				
Yes	1.55 (0.82– 2.93)	0.178	1.38 (0.70–2.72)	0.346
No	Ref		ref	
**Number of sexual partners in the past 6 months**				
≥ 1	1.35 (0.86–2.12)	0.192	1.19 (0.74–1.93)	0.478
< 1	Ref		ref	

*9v-HPV, nine-valent HPV types, including HPV6, 11, 16, 18, 31, 33, 45, 52, and 58; HR, Hazard Ratio; ref, reference category*.

### Sensitivity Analyses

After a conservative definition of clearance, we analyzed the reinfection rate of any 9v-HPV, any 4v-HPV, any 2v-HPV, and type-specific HPV. The conservative reinfection rate of any 9v-HPV was 13.09 per 1,000 person-months (95% *CI*: 8.53–19.05). Except for HPV45 which no conservative reinfection was observed, the rate of conservative reinfection for the other 8 types ranged from 6.57 per 1,000 person-months for HPV18 (95% *CI*: 1.09–20.28) to 23.04 per 1,000 person-months for HPV52 (95% *CI*: 10.53–42.89). For most HPV types, the rate of conservative reinfection was higher than reinfection (Table 4).

**Table 4:** Incidence rates^*^ of reinfection and conservative reinfection of specific HPV types among HIV-negative MSM (*N* = 515).

**Type**	***n***	**Reinfection**			***n***	**Conservative Reinfection**		
		**Reinfected number** **(%)**	**Person-months at risk**	**Rate** **(95% CI)**		**Reinfected number** **(%)**	**Person-months at risk**	**Rate** **(95% CI)**
9v-HPV	289	80 (27.68)	5,529.77	14.47 (11.52–17.87)	186	24 (12.90)	1,833.43	13.09 (8.53–19.05)
4v-HPV	254	55 (21.65)	5,101.48	10.78 (8.18–13.89)	176	20 (11.36)	1,613.70	12.39 (7.72–18.65)
2v-HPV	136	11 (8.09)	3,151.95	3.49 (1.81–5.98)	99	7 (7.07)	1,015.21	6.90 (2.96–13.33)
HPV6	135	26 (19.26)	2,697.57	9.64 (6.39–13.83)	97	8 (8.25)	881.52	9.08 (4.15–16.89)
HPV11	84	19 (22.62)	1,686.14	11.27 (6.93–17.12)	63	8 (12.70)	610.36	13.11 (5.99–24.40)
HPV16	103	25 (24.27)	2,159.22	11.58 (7.61–16.73)	76	13 (17.11)	760.30	17.10 (9.41–28.15)
HPV18	54	6 (11.11)	1,213.17	4.95 (1.97–10.02)	39	2 (5.13)	304.31	6.57 (1.09–20.28)
HPV31	48	10 (20.83)	1,085.45	9.21 (4.62–16.16)	37	4 (10.81)	313.14	12.77 (3.96–29.67)
HPV33	47	10 (21.28)	975.01	10.26 (5.14–17.99)	35	6 (17.14)	424.50	14.13 (5.62–28.64)
HPV45	27	1 (3.70)	629.57	1.59 (0.09–6.99)	16	0 (0.00)	132.42	/
HPV52	73	23 (31.51)	1,280.80	17.96 (11.58–26.33)	40	8 (20.00)	347.15	23.04 (10.53–42.89)
HPV58	78	22 (28.21)	1,470.49	14.96 (9.55–22.11)	52	6 (11.54)	547.60	10.96 (4.35–22.20)

* *Per 1,000 person-months*. *MSM, men who have sex with men; HIV, human immunodeficiency virus; HPV, human papillomavirus; CI, confidence interval; 9v-HPV, nine-valent HPV types, including HPV6, 11, 16, 18, 31, 33, 45, 52, and 58; 4v-HPV, quadrivalent HPV types, including HPV6, 11, 16, and 18, and 2v-HPV, bivalent HPV types, including HPV16 and HPV18*.

## Discussion

Our results showed that 27.68% of HIV-negative unvaccinated MSM will be reinfected after natural clearance of a specific HPV type, and the reinfection rate of any 9v-HPV was 14.47 per 1,000 person-months (95% *CI*: 11.52–17.87). HPV52 had the highest reinfection incidence rate of all specific HPV types. HPV6 and HPV16 had a higher number of reinfected individuals compared to other types.

There are fewer studies on anal HPV reinfection in MSM, which limits the comparison of our findings with other studies. Only one study on genital recurrence in men found that the recurrence of HPV infections was 20%, and HPV types 58, 52, 18, and 16 had the highest rates of recurrence (18). Similar to our study, the reinfection of 9v-HPV was 27.68%, and HPV52 had the highest reinfection rate. Reinfections of HPV were also reported in the few studies conducted among women. In a study by Krings et al. among women, 21.2% had acquired new high-risk infections with other genotypes after clearance of the original infection (19). A study by Moscicki et al. observed redetection of HPV-16 DNA in 18.1% of women (14). In a study of young women, 19.4% of incident infections of multiple genotypes were redetected within a year (15). However, in our study, the reinfection rates of specific HPV types were higher than in adult women (16). It is important to note that previous studies on the prevalence of HPV infection vary in different populations and geographical regions, such as the prevalence of HPV infection being higher in HIV-negative men than in HIV-negative women (3), making comparisons between studies difficult.

A study found that rather than inducing protective immunity, HPV infection strongly increases the risk of a future infection by the same type, by investigating HPV transmission dynamics by fitting mechanistic models to data from unvaccinated men (20). Research of Sofifie H. Mooij also shows no evidence for a protective effect of naturally induced HPV antibodies on subsequent anogenital HPV infection in MSM (13). As men appear not to develop immunity to HPV following natural infection, the reinfected HPV type will likely depend on the prevalence of the individual HPV types circulating in the community. Studies have reported that the most common high-risk HPV type among HIV-negative MSM were HPV52, 58, 16, and 18 (5, 6, 21–24). Therefore, it is understandable that HPV types 58, 52, and 16 had the highest rates of reinfection in our study. Since HPV16 is the most common HPV type associated with cancers (25, 26), the result of the highest number of HPV16 reinfected individuals in our study should be noted. Vaccine efficacy trials have shown that the vaccination of children before the first sex can significantly reduce the infection rate, and vaccination of previously infected individuals can also reduce the prevalence of HPV (27–30). Thus, early vaccination of MSM with the HPV vaccine is important to prevent HPV prevalence.

Moreover, the risk of HPV infection depends on differences in demographic and behavioral risk factors between populations. For example, HIV positivity and a higher number of sexual partners can increase the rate of HPV infection and persistent infection (31–34). Patterns of anal intercourse, a history of condom use during homosexual behaviors, and drug use were reported to be the main factors influencing HPV infection (35, 36). In our study, univariate analysis showed that MSM over the age of 30 had a slightly higher risk of reinfection with any 9v-HPV, but no significant association was observed in multivariate analysis. Epidemiological studies worldwide have found two peaks of HPV infection in women. The first occurs a few years after sexual debut, and the second peak occurs around the time of menopause (37). There are two main theories to explain this occurrence: (1) a new sexual partner or an increased number of sexual partners; and (2) reactivation of latent infections acquired many years earlier (38). In earlier studies, the prevalence of HPV among MSM did not reveal an obvious peak phenomenon. In contrast, a study showed that seroprevalence increased with age among young-to-middle-aged men with significant upward age trends observed for HPV 11, 16, and 18 (39). This finding may be related to the number of new sexual partners. With an increase in age, the proportion of women with more than one sexual partner will decline, whereas the proportion of MSM remains at a relatively stable level (40). Unfortunately, there are currently not enough numbers in our study to do an analysis by age. With the implementation of our cohort study, we should understand the relationship between the number of sexual partners and HPV reinfection in different age groups, to further clarify the true situation of age, the number of sexual partners, and HPV reinfection.

## Strengths and Limitations

To our knowledge, this is the first study to characterize the reinfection of anal HPV among HIV-negative MSM in China. Important limitations associated with this study should be recognized. First, we did not distinguish between HPV reactivation and reinfection. In our future studies, we can use next-generation sequencing to shed light on the origin of the reinfections. Second, the negative results of a subsequent sample after infection were considered to indicate cleared, without considering that the DNA content in the sample may be below the threshold of detectability, which may increase the number of events cleared. However, we still found similar results after using the conservative definition of clearance, which improves the credibility of this research results. We must also be aware of the impact of a small number of reinfection events on the stability of results.

## Conclusion

In conclusion, our study found a high rate of anal 9v-HPV reinfections among HIV-negative unvaccinated MSM. Therefore, HPV vaccination is recommended not only prior to sexual debut but also after viral clearance for MSM to reduce HPV prevalence. Moreover, future studies are needed to understand the role of reinfection in the etiology of HPV-associated diseases.

## Data Availability Statement

The original contributions presented in the study are included in the article, further inquiries can be directed to the corresponding author.

## Ethics Statement

The studies involving human participants were reviewed and approved by the Ethics Committee of the First Affiliated Hospital of Xinjiang Medical University (20160512-11). The patients/participants provided their written informed consent to participate in this study.

## Author Contributions

LL and ZZ: data curation. LL, GZ, ZZ, and LW: material preparation and data collection. LL and GZ: analyzed and interpreted the data. LL: original draft of the manuscript. JD and GZ: funding acquisition. DW: polished and reviewed the language of the main article. All authors have read and agreed to the published version of the manuscript.

## Funding

This research was funded by the Natural Science Foundation of China (Grant Numbers 81560539 and 81860590) and the Natural Science Foundation of Xinjiang Uygur Autonomous Region (Grant Number 2019D01C204).

## Conflict of Interest

The authors declare that the research was conducted in the absence of any commercial or financial relationships that could be construed as a potential conflict of interest.

## Publisher's Note

All claims expressed in this article are solely those of the authors and do not necessarily represent those of their affiliated organizations, or those of the publisher, the editors and the reviewers. Any product that may be evaluated in this article, or claim that may be made by its manufacturer, is not guaranteed or endorsed by the publisher.
